# Ectoparasite prevalence in farmed seabass (*Dicentrarchus labrax*) from Damietta, Egypt: environmental correlations and histological consequences

**DOI:** 10.1007/s11259-026-11084-9

**Published:** 2026-02-21

**Authors:** Mohamed Arafat, Zeinab Hassan, Basem Elmishmishy, Viola H. Zaki, Eman Zahran

**Affiliations:** 1 Department of Aquatic Animal Medicine, Faculty of Veterinary Medicine, Mansoura University, Mansoura, 35516 Egypt; 2 Horus Research Center, Horus University-Egypt (HUE), Coastal Road, New–Damietta, Egypt; 3 Fish Disease Department, Faculty of Veterinary Medicine, Aswan University, Aswan, 81528 Egypt; 4 Department of Parasitology, Faculty of Veterinary Medicine, Mansoura University, Mansoura, 35516 Egypt

**Keywords:** Mariculture, Ectoparasite, Seasonal prevalence, European seabass, Water quality, Heavy metals, Histopathology, SEM

## Abstract

**Background:**

Egypt's mariculture industry relies heavily on the seabass sector, a major marine fish species in Mediterranean aquaculture. However, industries face significant challenges such as disease outbreaks, aquatic pollution, and poor water quality.

**Methods:**

This investigation focused on the parasitism of seabass *(Dicentrarchus labrax)* in the Damietta Governorate of Egypt, during the period from August 2022 to July 2023. A total of 200 seabass specimens, weighing 25–200 g was collected through random sampling. The fish samples underwent a series of assessments, including clinical evaluations, parasitological investigations, and histopathological analyses. Concurrently with fish collection, various water parameters have been documented, including temperature, salinity, pH, and heavy metal concentration.

**Results:**

Parasitological examination revealed a high prevalence (75%) of ectoparasitic protozoa belonging to *Trichodina spp.* The ectoparasitic copepod *Caligus spp.* had a total prevalence of 15%, whereas a single Isopoda species had an infestation rate of 2%. The chi-square trend analysis revealed a significant positive relationship between *Trichodina* prevalence and seasonal changes (*P* < 0.001). The water parameters tested showed a significant negative relationship between *Trichodina* prevalence and water temperature (*r* = -0.99), with a non-significant negative relationship with water salinity (*r* = -0.75). The heavy metal levels across seasons were higher than the permissible limits. Histopathological changes in infected tissues indicated various types and degrees of lesions, and Scanning Electron Microscopy (SEM) observations highlighted the parasite-host relationship due to parasitic infestations.

**Conclusion:**

Overall, abiotic stressors could be responsible for the greater prevalence of parasitic infestations in fish, negatively affecting tissue structure and fish health. Therefore, the strict monitoring of fish farms and biosecurity practices must be implemented to guarantee fish welfare and sustainability.

## Introduction

In Egypt, aquaculture is essential for rural development and food production, especially in coastal areas with few employment options. The industry is known for its rapid expansion and its ability to improve food security (Mehrim and Refaey 2023). Mariculture mainly produces many fish, such as mullet *(Mugil cephalus; Liza ramada)*, European sea bass *(D. labrax),* and gilthead sea bream *(Sparus auratus)* in northern Egypt, especially in the areas of Damietta, Port Said, Alexandria, and the Suez Canal. It is considered a significant industry contributing to the economy (Shaalan et al. 2018). Egypt's mariculture industry, regarded as one of the top producers in the Mediterranean, depends heavily on the seabass industry, which is attributed to the favorable conditions that enable the production of quality marine fish with a sizable market, such as competitive prices, good weather, and wide aquaculture sites (Abdel-Hady et al. 2024). However, this industry inevitably faces many challenges such as disease outbreaks, water pollution, poor water quality, and climate change (Goddek et al. 2019).

Fish are extremely vulnerable to numerous diseases (bacterial, fungal, viral, and parasitic), particularly when raised in uncontrolled environments (Kalaria et al. 2024). It significantly affects mariculture growth, fertility, mortality rate, and marketability (Bula et al. 2023). The most problematic challenges are parasitic infestations affecting fish, including seabasses, such as *Trichodina* species, and crustaceans, such as *Caligus* (sea lice) and Isopoda species (Eissa et al. 2020). Abiotic stressors, such as poor water quality, heavy metal pollution, and serious ecological hazards globally, particularly in Egypt, compromise fish immunity and render fish susceptible to pathogens, including parasitic infestations (Radwan et al. 2022; Abbas et al. 2024).

Climate change is a global issue that affects mariculture (Danylchuk et al. 2023), aquaculture sustainability and production by modifying ecosystem dynamics and water quality, and interacting with anthropogenic impacts, including water management techniques such as the discharge of pollutants and drainage (Maar et al. 2024; Matolia et al. 2024). Fluctuations in temperature, salinity, pH, and dissolved oxygen, along with increased disease prevalence, altered species composition, and reduced water quality (Matolia et al. 2024), negatively affect fish health (Little et al. 2020). Variations in water temperature generally affect seabass health, as well as the frequency of ectoparasitic infestations, as the prevalence of ectoparasites can be very high in wild and cultured fish populations due to fluctuations in temperature (Gehman et al. 2018). Studies by Jerônimo et al. (2022) indicated that ectoparasites thrive under specific salinity ranges, with marine species such as *Caligus,* being particularly prevalent at higher salinities, whereas freshwater species struggle under brackish conditions. Additionally, the prevalence of *Trichodina* and Isopoda has been positively correlated with elevated temperatures and nitrite levels, suggesting that deteriorating water quality exacerbates parasitic infestations (Mahmoud et al. 2019).

Histopathological changes have been widely used as biomarkers to evaluate the health of fish exposed to contaminants and infestations (Ahmed et al. 2019). Histopathology can be used as an indicator of several abnormal circumstances in the fish ecosystem (environment) and provides a quicker way to detect the impact of pathogens (such as protozoan parasites) on various fish organs, including the gills (Naiel et al. 2019; Ahmed et al. 2020). Additionally, electron microscopy (scanning electron microscope, SEM) has revealed detailed morphological characteristics of these parasites and gill tissues, enhancing identification and understanding of their impact on fish health (Abd-ELrahman et al. 2023).

The current study aimed to investigate the ectoparasitic prevalence in farmed seabass (*Dicentrarchus labrax*) and their correlation with the ecological conditions, such as temperature, salinity, and heavy metal concentrations. Morphological identification using light microscopy and scanning electron microscopy (SEM) was employed to confirm the identity of the key ectoparasites. Additionally, histopathological examination of infected tissues was performed to assess the physiological impact of parasite infestations on host organs, thus strengthening the interpretation of infection severity. These approaches can inform early warning systems and enhance routine health surveillance during aquaculture operations.

## Materials and methods

### The study area

**Fig. 1:**
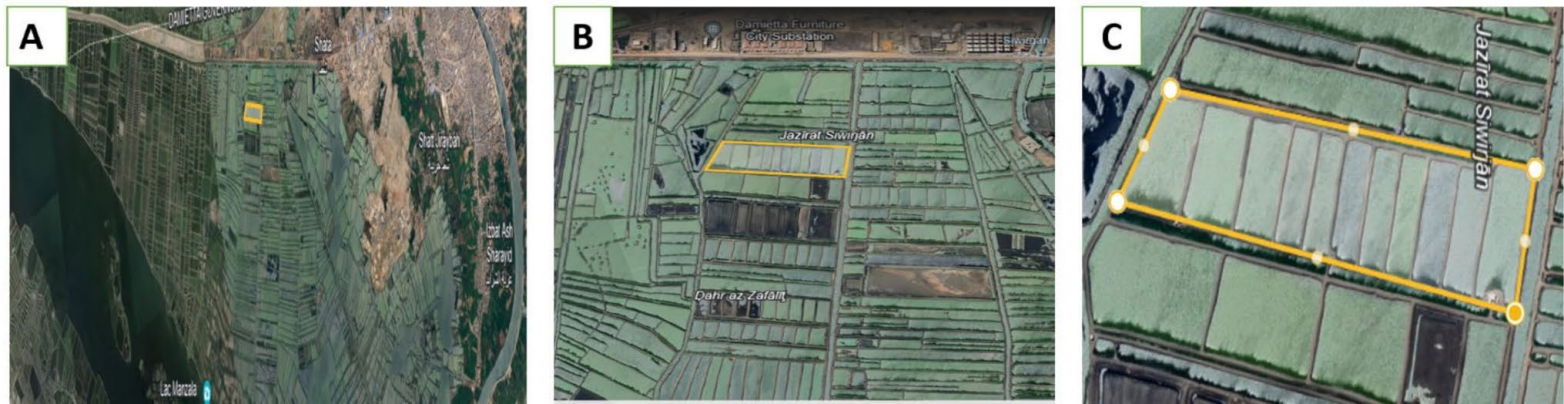
A map from google earth showing a sampling site in seabass farm, Damietta, Egypt https://earth.google.com/web/@31.41251675,31.89294375,0.11881887a,2026.42047876d,35y,−107.00773933h,1.77334048t,0r

The present study was conducted at a private fish farm located in the Shatta region of the Damietta Governorate in northeastern Egypt using water sourced from El Manzala Lake and the Mediterranean Sea. “31°24′32.9"N, 31°53′27.6"E.”, via Google Earth https://earth.google.com/web/@31.41251675,31.89294375,0.11881887a,2026.42047876d,35y,−107.00773933h,1.77334048t,0r. Figure 1, The farm inspections were conducted seasonally during the period from August 2022 to July 2023. A structured, personally administered questionnaire was administered via oral interviews, with minor modifications to collect data on the farm’s history and management practices (Ismail et al. 2024).

### Fish sampling

Two hundred specimens of European seabass, *D. labrax*, 50 fish per season (n = 200 total), were collected seasonally, with an average size of 25–200 g. The collected fish were transported alive in large thick plastic bags supplied with oxygen. Freshly dead fish samples were stored in isothermal boxes on ice and immediately transported to the Aquatic Animal Medicine Lab (AAML), Faculty of Veterinary Medicine, Mansoura University, Egypt. The fish were thoroughly examined externally and internally for clinical abnormalities (Noga 2010).

### Water sampling

A sterile 500 mL glass bottle was used to collect 500 mL of water “duplicate samples (*n* = 2)” per season, from multiple pond sites at a depth of 30 cm below the water surface. Each sample was marked with the required information. Samples were transported to the laboratory in an icebox and stored at 4 °C (Boyd and Tucker 2012). The physical parameters of the water, such as temperature, electrical conductivity (salinity), and pH, were measured in situ using portable meters (Lovibond®, Dortmund, Germany). Heavy metals such as Iron, Mercury, Zinc, Nickel, Cadmium, Arsenic, and Lead were measured twice per season using inductively coupled plasma (iCAP™ 7000 Plus Series ICP-OES, Thermos Scientific™) after acid digestion using HNO_3_ (69%) and HCl (30%) in a microwave digestion apparatus (Milestone MLS 1200) in the central laboratory of the Faculty of Agriculture, Mansoura University, Egypt, following the method of AOAC (1995). The limits of quantification (LOQ) under the present laboratory conditions were: Fe 0.005 mg/L, Zn 0.002 mg/L, Ni 0.005 mg/L, Cd 0.001 mg/L, As 0.005 mg/L, Pb 0.005 mg/L, and Hg 0.001 mg/L.

### Parasitological examination

The fish were examined externally for ectoparasites through wet-mount preparations of skin, fins, and gills. Briefly, the fish were scraped from just behind the operculum to the tip of the tail fin on both sides of the fish's body and gills with a cover slip and a scalpel blade. Mucus was transferred to slides and examined microscopically (Reavill and Roberts 2007). The slides were fixed with absolute methanol, air-dried, and stained with Giemsa (Sigma–Aldrich). The stained smears were washed for 15–20 min, left to dry, and examined carefully under a light microscope (CX31; Olympus, Japan) (Eissa et al. 2017a). All prepared slides were examined to identify parasite species. Protozoan parasites have been identified on the basis of their morphology (Woo and Buchmann 2012). The skin, eyes, gills, fins, opercula, and mouth cavities of the crustacean parasites were examined. The detected crustacean parasites were washed with physiological saline to remove mucus and debris, left in a refrigerator at 4 °C until complete relaxation, cleaned, preserved in lactophenol (Fleck and Moody 1988) and examined under a light microscope. The morphological features of the collected protozoan ectoparasites were based on a prior description of their morphology by Woo and Buchmann (2012), which confirmed species-level identification. At the same time, the identification of *Caligus spp.* and Isopoda was based on morphological criteria described in authoritative keys (Kabata, 1982, and Bruce 1986) as observable under light microscopy and scanning electron microscopy (SEM).

### Histopathological examination

The gills of infested live fish were subjected to histopathological examination. Tissue samples were fixed for 24 h in 10% phosphate-buffered formalin, dehydrated in a graded ethanol series, cleared in xylene, embedded in paraffin, sectioned at 5 mm, and stained with hematoxylin and eosin (H&E) according to Roberts (2012). The stained slides were examined under an Olympus CX31 light microscope (Olympus Corporation, Japan) equipped with Olympus DP20 Microscope Camera. Lesion severity has been semi-quantitatively described in the revised caption (e.g., % lamellae affected) based on approximate histopathological observations.

### Scanning electron microscopic (SEM) examination

For SEM, crustacean parasites and gill tissue samples, measuring 0.5–1 cm, were placed directly in a fixative solution containing 4% paraformaldehyde and 1% glutaraldehyde in a 0.1 M phosphate buffer (pH 7.2). The specimens underwent a rinsing procedure utilizing the buffer before being subjected to post-fixation in a 1% osmium tetroxide solution for 2 h at a temperature of 4 °C within the same buffer. The fixed specimens were subjected to dehydration and subsequently underwent critical point drying by systematically applying ascending ethanol concentrations of 30, 50, 70, 90, and 100%, each for 30 min, and then samples were dried in tetramethyl saline (TMS), according to Dey et al. (1989). Next, the samples were mounted on brass stubs and coated with gold–palladium ions using Denton Vacuum Desk II SEM sputter coater, as described by Dey et al. (1989), and observed using a Joel JSM-6510 L.V. SEM. The microscope was operated at 30 kV at the EM Unit, Faculty of Agriculture, Mansoura University, Egypt. The obtained images were taken by CCD digital camera (Model XR- 41) and analyzed using ImageJ software.

### Statistical analysis

All statistical analyses were performed and visualized using GraphPad Prism 8 (GraphPad Software, San Diego, CA, USA). Data were analyzed using a simple descriptive statistical analysis to calculate the prevalence of parasitic infestations. The following formula was applied: Prevalence% = (number of infected hosts/total number of hosts examined) × 100. Differences among months and seasons were calculated using the chi-square test, and a P value < 0.05 was considered significant. Trends in parasite prevalence across ordered seasons (Summer → Autumn → Spring → Winter) were analyzed using the Chi-square test for trend (Cochran–Armitage test), which evaluates the presence of a significant linear trend in proportions across ordered groups. The ecological parameters were analyzed using one-way ANOVA. Post-hoc analysis was performed using Tukey's test, and Kolmogorov–Smirnov (distance) and Shapiro–Wilk tests were applied before checking the normality and homogeneity of the samples. Parametric correlation analysis was performed to establish the degree of correlation between the ectoparasitic prevalence and water parameters (temperature and salinity), and 95% confidence intervals (CI) were computed for each correlation. Correlation analyses were performed using the mean seasonal water temperature and salinity values against the total number of *Trichodina* parasites recorded per season. Because each point represents aggregated seasonal data, no error bars were plotted. The Pearson’s correlation coefficient (r) was considered statistically significant at *P* < 0.05.

## Results

### Criteria of farm facility and cultured fish

The following data were obtained from a questionnaire that assessed the management procedures and history of the fish farms (Table 1). The farm owner was 49 years old, had a moderate level of education, and was employed on the farm for almost 20 years. The farm was located in the Shatta region of Damietta Province, Egypt (Fig. 1), and employed a semi-intensive production system with water provided from Lake Manzala and the Mediterranean Sea. The farm utilized feed supplements from a specialized feed store and allowed for everyday water exchange. Chemical products, such as disinfectants (hydrogen peroxide, “H_2_O_2_” and formalin) and veterinary drugs, are only used when necessary for disease control. The fish production rate ranged from 2 to 3 tons per feddan, with marketing weights ranging from 400 to 500 g; however, in some cases, fish may have exceeded 500 g at the market size.

**Table 1:** Questionnaire pertained to the farm management practices and the cultured fish

Inquiry	Provided data
About owner	
• Age	49
• Gender	male
• Education level	middle education level
• Experience level	20 years
About the farm	
• Farm location	Shatta area, Damietta
• Type of production	Semi-intensive
• Farm size (feddan/m^2^)	17 feddan
• Production method	Earth ponds
• Fish stocking density/feddan	5000 fish/feddan
• Water source of the farm	Lake of Manzala and the Mediterranean Sea
• Water exchange rate/pond	Every other day
• Quality of pond water	Slight turbidity
• Disinfectants used	H_2_O_2_ and Formalin
• Veterinary supervision (Yes/NO)	No
• Use of untreated poultry manure	No
• How dispose-off dead fish	**Buried**
• Have special food store (Yes/No)	Yes
About farmed fish (seabass)	
• Fish mortality rate	Sporadic cases
• Mortality rate in the last 2 years	Not evident
• Fish production rate	2 to 3 tons/feddan
• Marketing weight (g)	400 to 500 g/Fish

1 feddan = 0.42 hectare (ha)

### Water quality parameters and heavy metals assessment

Water parameters (pH, **t**emperature, and **s**alinity) were recorded during the different seasons on the farm (Table 2). Salinity (g/L) showed a significant increase (*P* < 0.05) in Winter (49.4 ± 0.68) compared to spring (41.3 ± 0.69), summer (37.4 ± 0.50), and autumn (29.3 ± 0.61). Temperature (°C) showed a significant increase (*P* < 0.05) in summer (26.30 ± 0.33) compared to other seasons, winter (20.33 ± 1.02), autumn (23.33 ± 0.44), and spring (21.33 ± 0.45). The pH values showed a significant increase (*P* < 0.05) in Winter (8.60 ± 0.06) compared to spring (8.0 ± 0.05) and summer (8.10 ± 0.05), without statistical changes compared to autumn (8.33 ± 0.09). The seasonal concentrations of heavy metals (mg/L), including Hg, Cd, Fe, Pb, Zn, As, and Cu, were higher than permissible limits and are summarized in (Table 3).

**Table 2:** Physical parameters of the water during different seasons

Water parameters		**Season**		
	Summer	Autumn	Spring	Winter
Temperature(°C)	26.30^a^ ± 0.33	23.33^b^ ± 0.44	21.33^bc^ ± 0.45	20.33^c^ ± 1.02
Salinity (g/L)	37.4**0**^c^ ± 0.50	29.30^d^ ± 0.61	41.3**0**^b^ ± 0.69	49.4**0**^a^ ± 0.68
pH	8.10^bc^ ± 0.05	8.33^ab^ ± 0.09	8.00^c^ ± 0.05	8.60^a^ ± 0.06

^*^All data are expressed as mean ± SEM (*n* = 3). Different superscript letters on the mean values in a column indicate statistically significant differences (*P* < 0.05)

**Table 3:** Heavy metals assessment (mg/L) in the farm in different seasons

Heavy Metals(mg/L)	Seasons				(USEPA, 2000)^a^	Law 48/1982^b^	FAO (2017)^c^
	Summer	Autumn	Spring	Winter			
Hg	0.085	0.111	2.379	0.212	**-**	0.001	**-**
Cd	0.136	0.302	0.063	ND	0.005	0.01	0.01
Fe	8.968	10.861	3.284	8.055	0.01	< 1	** < 0.3 mg**
Pb	1.785	2.486	ND	1.872	0.02	0.05	** < 0.01**
Zn	5.624	3.525	0.186	5.110	0.005	< 1	** < 0.03**
Cu	2.470	0.588	ND	0.17	0.05	-	-
As	N.D	0.087	1.269	N.D	0.03	1	0.2

^a^ USEPA (United States Environmental Protection Agency (USEPA) 2000. Risk-Based Concentration Table. United States Environmental Protection Agency, Philadelphia, PA USA

^b^ Law 48/1982: Egyptian Law for Protection of the River Nile and Waterways from Pollution, Art. (60) Water quality in the River Nile

^c^ FAO: FAO 2017. Aquaculture Regional Reviews. In: FAO Fisheries and Aquaculture Department. Rome

*N.D* not detected

### Clinical signs

**Fig. 2:**
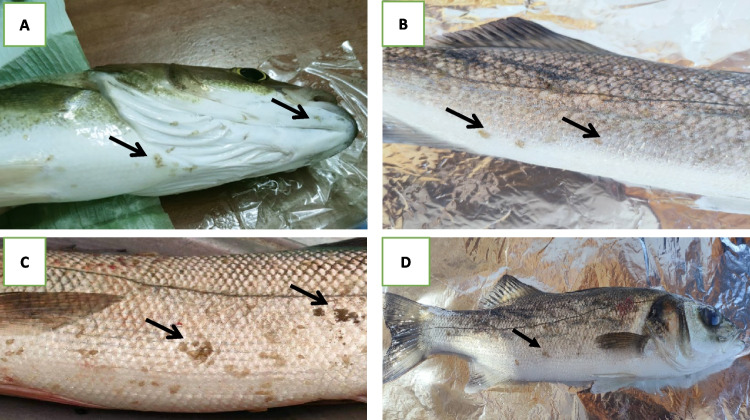
**A, B, C** Excessive ectoparasitic infestation with *Caligus* species on the head and abdominal areas, **D** showing ectoparasitic infestation with *Caligus* species and an area of redness and inflammation on the skin

**Fig. 3:**
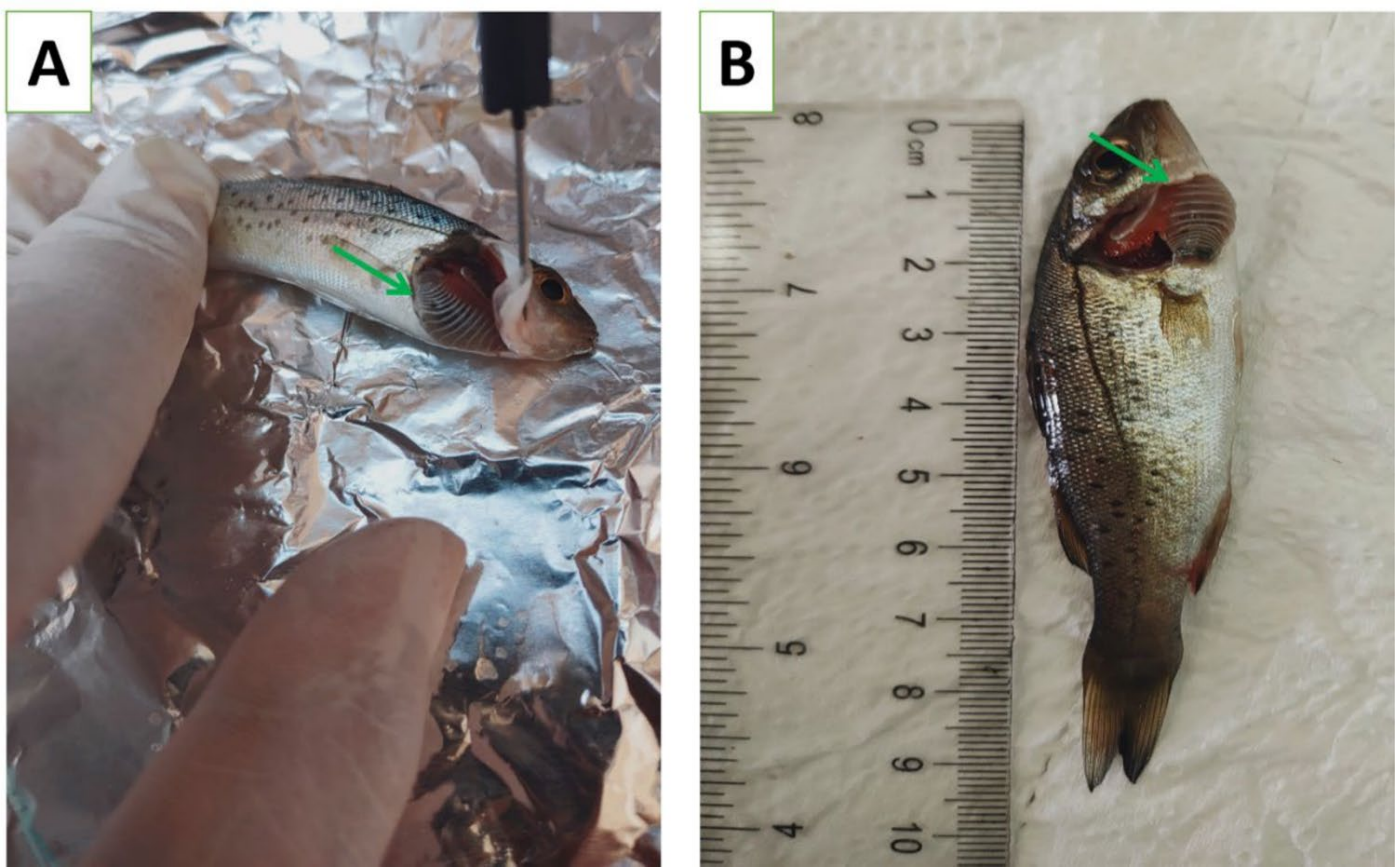
**A, B** Isopoda species attached to the fish's gills with excessive mucus secretions

Clinical examination of naturally infested sea bass fish revealed no noticeable clinical abnormalities, except in cases of heavy infestation with crustacean ectoparasites. Some infested fish exhibited respiratory signs and swimming with open mouth. The other fish showed a hemorrhagic area in the abdomen. In some cases, *Caligus* species (Fig. 2 A-D) and Isopoda species (Fig. 3 A&B) were observed on the gills and body surface, respectively. The infected gills were covered with mucus. Heavily infested body surfaces exhibit excessive mucus secretion and redness of the skin.

### Parasite examination, identification, and morphological description

**Fig. 4:**
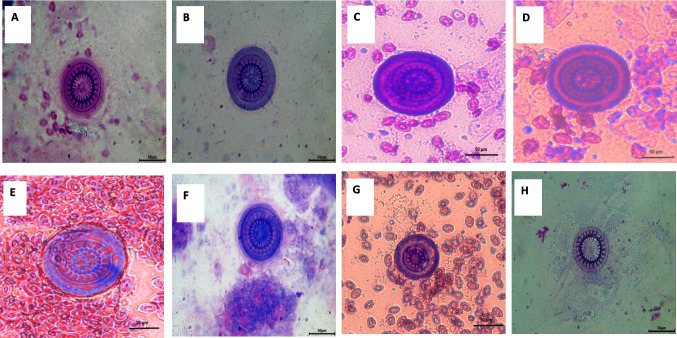
Giemsa-stained wet mounts showing presumptive *Trichodina* spp. recovered from the gills of European seabass (*Dicentrarchus labrax*): **A** morphologically resembling *T. fultoni*, **B** *T. heterodentata*, **C** *T. californica*, **D** *T. compacta*, **E** *T. reticulata*, **F** *T. nobilis*, **G** *T. nigra*, and **H** *T. rectuncinata*. These identifications are tentative and based solely on external morphology under 100 × magnification using Giemsa stain; no silver impregnation or denticle ring visualization was performed, and therefore species-level identification was not confirmed. Scale bar = 50 µm

**Fig. 5:**
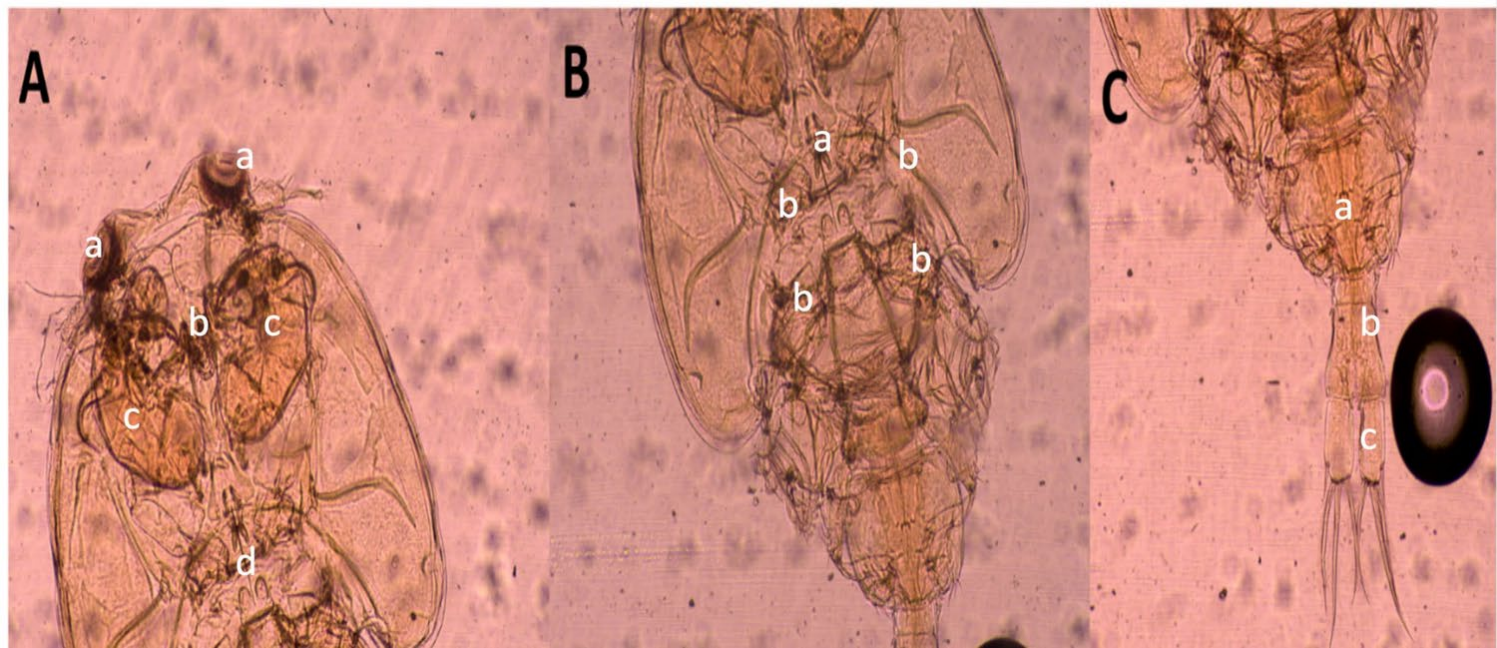
Light microscopic micrograph of the parasite *Caligus* spp. **A**, **B** The whole parasite, which is oval-shaped, comprises the cephalothoracic shield. A: The cephalic area contained a well-developed frontal plate with disk-shaped lunules (a), mouth cone(b), and maxillae(c) 2 swellings (d). **B** showing (a) sternal furca (b) the legs and their hair. **C** The posterior part of the parasite (a) gonadal segment, (b) abdominal segment (c) caudal lamella

**Fig. 6:**
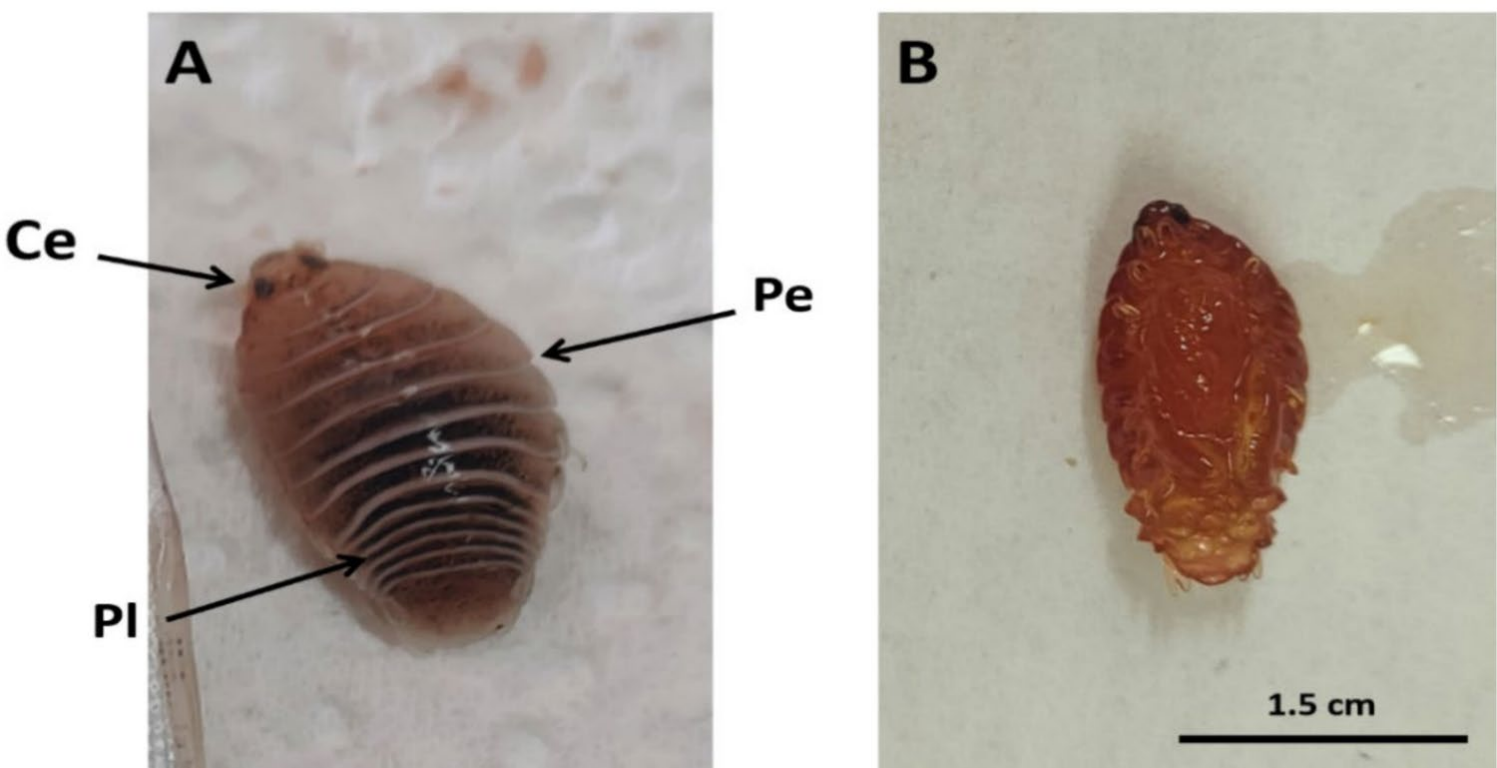
**A** Dorsal view and** B** Ventral view of a presumptive *Livoneca redmanii* (Leach, 1818) (Isopoda) isolated from the body surface of European seabass (*Dicentrarchus labrax*). Identification was based on external morphological features consistent with descriptions in Bruce (1986). (Ce) cephalon; (Pe) pereon; (Pl) pleon. Scale bar = 1.5 cm

Morphological identification of ectoparasites was carried out using light microscopy by applying standard taxonomic criteria from well-established references. This step was essential for determining parasite presence and estimating prevalence across sampling points. Out Of 200 examined fish, 154 were infested with different ectoparasites, with a total infestation rate of 77% (Table 4). External parasitological examination revealed a high prevalence (75%) of ectoparasitic protozoa belonging to *Trichodina spp.* (Fig. 4 A-H). A comparative reference morphometric data (Table 5) based on Woo and Buchmann (2012), intended purely for comparative and illustrative purposes. These comparisons are tentative and not diagnostic. *Caligus spp*. An oval, flattened body, shield-like cephalothorax with lunules, four pairs of legs, segmented genital and abdominal regions, and clear body segmentation (Fig. 5). Isopoda, *Lepeophtheirus redmanii* (Leach, 1818) has an ovoid, light brown body with black chromatophores, no median cephalic projection, and visible eyes (Fig. 6 A-B).

**Table 4:** Seasonal prevalence of ectoparasites* (*Trichodina* spp., *Isopoda* spp., and *Caligus* spp.) isolated from European seabass (*Dicentrarchus labrax*)

Season	Infested	*Trichodina* spp	*Caligus* spp	*Isopoda* spp
Summer	48% (24/50)	48% (24/50)	**0%**	8% (4/50)
Autumn	72% (36/50)	72% (36/50)	**0%**	**0%**
Spring	88% (44/50)	88% (44/50)	**0%**	**0%**
Winter	100% (50/50)	92% (46/50)	60% (30/50)	**0%**
Total	77% (154/200)	75% (150/200)	15% (30/200)	2% (4/200)

^*^All identifications are at the genus level except where species assignment could be supported with appropriate morphological criteria and references (e.g., *Livoneca redmanii*). For *Trichodina*, identifications were restricted to the genus level based on Giemsa-stained light microscopy

**Table 5:** Morphological features of the observed *Trichodina* spp. were compared to published descriptions in Woo & Buchmann (2012)

*Species	Body Diameter (µm)	Adhesive Disc Diameter (µm)	Denticular Ring Diameter (µm)	No. of Denticles	No. of Radial Pins/Denticle	Ray Description	Blade Description
*Trichodina fultoni*	51–76 (mean 63)	38–61 (mean 50)	32–38 (mean 35)	20–26	8–12	Stout, curved posteriorly, pointed tip	Curved, rounded tip, unattached
*Trichodina heterodentata*	73–91 (mean 82)	51–65 (mean 58)	34–40 (mean 37)	20–24	8–12	Straight, sharp tip	Sickle-shaped, rounded tip
*Trichodina californica*	43–49 (mean 46)	36–43 (mean 40)	21–25 (mean 23)	21–28	8–10	Short, straight, sharp tip	Bluntly rounded tip
*Trichodina compacta*	42–71	35–60 (mean 47)	21–34 (mean 28)	18–20	8–10	Short, stout, slightly curved	Cup-shaped, cogwheel appearance
*Trichodina reticulata*	44–68 (mean 55)	36–55 (mean 45)	27–36 (mean 31)	25–31	8–10	Thick, round tip	Angular, semilunar curve
*Trichodina nobilis*	67–86 (mean 77)	50 −76 (mean 65)	37 −56 (mean 44)	25–28	12–14	Long, straight to slightly curved	Curved with deep semi-lunar margin
*Trichodina nigra*	30–50	32–65	19–39	12–30	12–19	Not specified	Crescent-shaped, radial striations
*Trichodina rectuncinata*	35–55	29–43	15–21	14–32	10–12	Not specified	Crescent-shaped, radial striations

^*^ These data are tentative and not diagnostic

The current study did not perform morphometric measurements or species-level identification

### Seasonal prevalence of the recovered parasite in naturally infested seabass and its correlation to water parameters (temperatures and salinity):

**Fig. 7:**
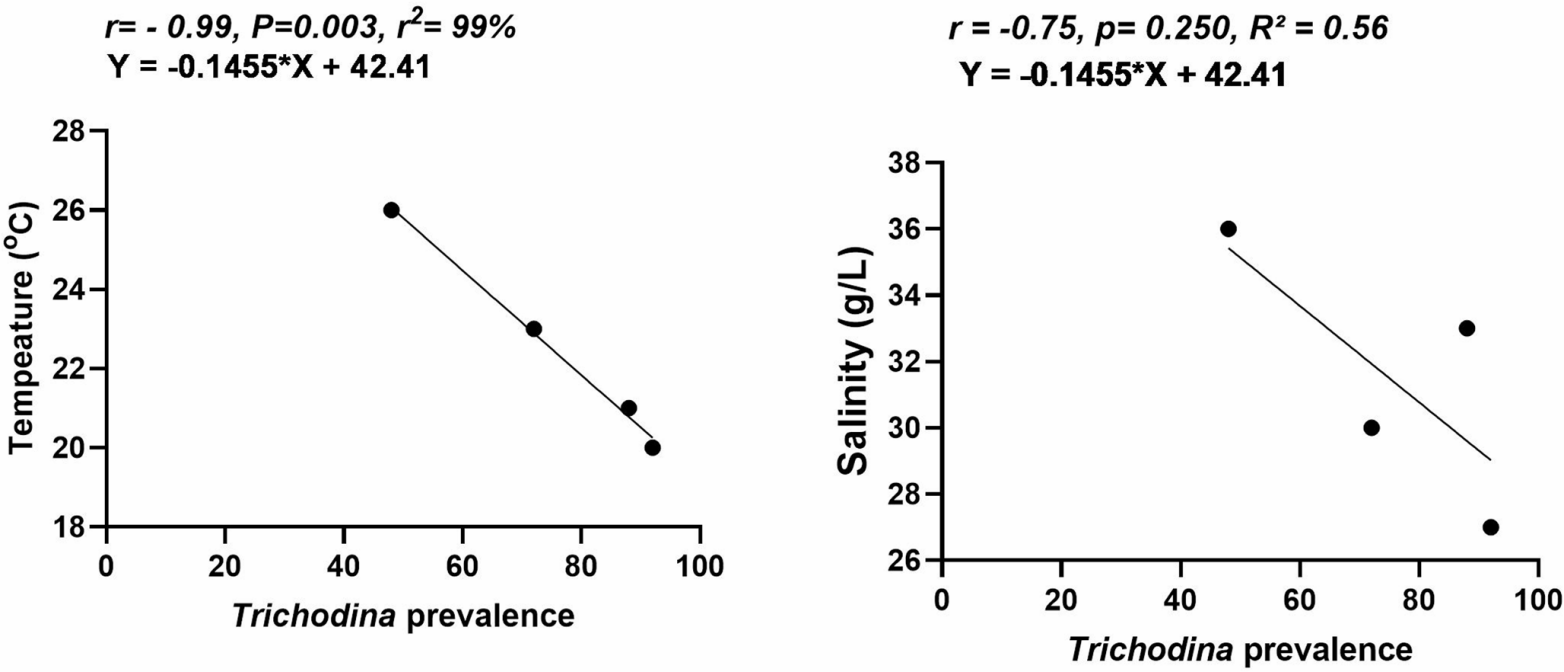
Correlation between *Trichodina* prevalence and two environmental parameters (Fig 7**A**. Temperature and 7**B**. Salinity) in different seasons. Trendlines indicate linear regressions, and corresponding equations and correlation coefficients (r) are shown

The prevalence of *Trichodina species* differed significantly between seasons (Chi-square, degree of freedom (df) = 3, *P* = 0.003). For *Trichodina* species, winter had the highest occurrence (92%), followed by spring (88%), autumn (72%), and summer (48%). Additionally, seasonal trends using the Chi-square test for trend (Chi-square, df = 1, *P* = 0.0001), were significantly increased across ordered groups (seasons arranged as Summer → Autumn → Spring → Winter) (Table 6). A trend was observed for *Caligus* species, which was only detected in winter, when the prevalence peaked at 60%. A similar trend was found for Isopoda species, which were only detected in the summer (8%). Statistical analyses could not be performed for Isopoda and *Caligus species* across different seasons because of their complete absence at different times during the sampling periods. Correlation analysis revealed a strong, insignificant negative correlation with water salinity (*r* = −0.75, *P* = 0.25, *R*^2^ = 0.562) and a strongly significant negative relationship between *Trichodina* prevalence and water temperature (*r* = −0.997, *P* = 0.003, *R*^2^ = 99%). Additionally, the chi-square test for trends (*P* < 0.0001) confirmed that *Trichodina’s* seasonal increase was statistically significant, aligning with the temperature correlation (Fig. 7).

**Table 6:** Seasonal prevalence of *Trichodina species* isolated from the fish

Seasonal prevalence of *Trichodina spp* infested seabass			
Season	*Trichodina spp*	Chi-square *p*-value	Chi-square test for trend *p*-value
Summer	48% (24/50)	0.003	0.0001
Autumn	72% (36/50)		
Spring	88% (44/50)		
Winter	92% (46/50)

### Histopathological examination

**Fig. 8:**
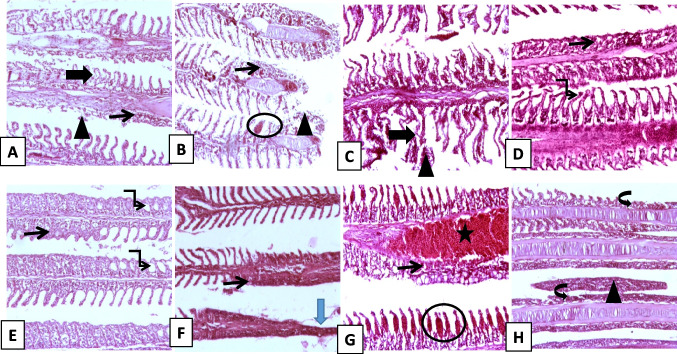
Histopathological changes in gill tissue (H&E, Image magnification = 100x, inset = 400x.) **A**–**B** Summer: gills show lamellar lifting with partial lamellar fusion, desquamated lamellar epithelial cells, and interlamellar necrotic debris, together with few lamellar telangiectases (affecting ≈ 25–30% of lamellae). **C**–**D** Winter: gills showing lamellar clubbing with lamellar necrosis and desquamation or coalescing lamellar hyperplasia, with fusion along the lengths of some lamellae or at lamellar tips forming synechiae (≈ 40% of lamellae affected). **E**–**F** Spring: focal to diffuse coalescing hyperplasia of lamellar epithelium causing fusion of secondary lamellae and partial loss of lamellar epithelium (≈ 35%). **G**–**H** Autumn: gill raker showing thickening and expansion of lamellar tissue with abundant lamellar telangiectasia and severe branchial congestion; lamellar fusion with goblet cell hyperplasia (≈ 45%). Additionally, diffuse marked distortion of normal gill architecture was characterized by stunted, fused, or completely lost secondary lamellae with massive interlamellar detachment of gill epithelium admixed with cellular infiltrates. Thin arrow = lamellar fusion; thick arrow = lamellar clubbing and lifting; twisted arrow = synechiae formation; arrowhead = necrotic or detached gill epithelium; circle = lamellar telangiectasis; star = branchial congestion; curved arrow = stunted, fused epithelium with complete loss of secondary lamellar architecture; blue arrow = complete loss of lamellar epithelium

**Fig. 9:**
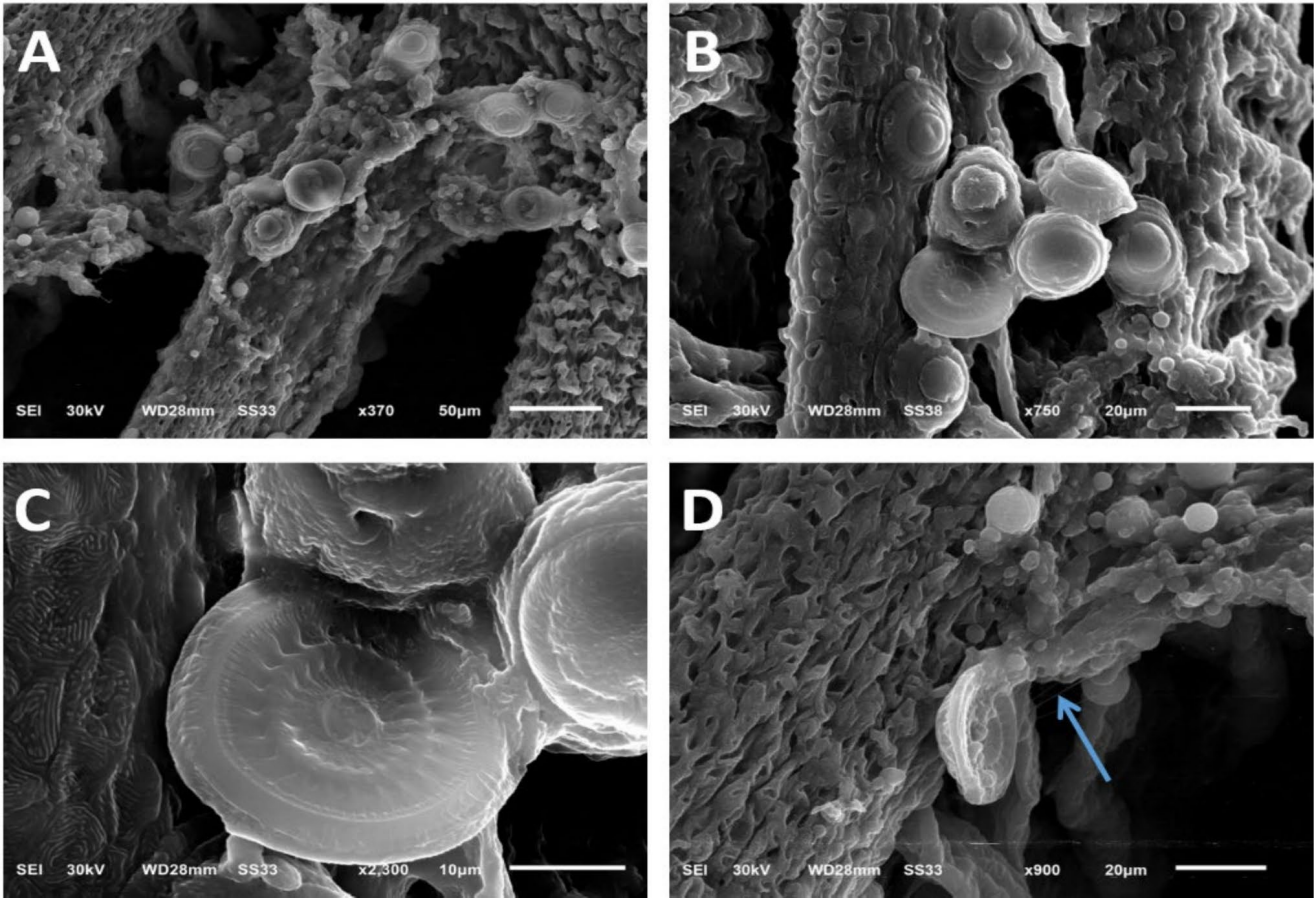
Scanning Electron Microscopy (SEM) image of gills showing parasite-host interaction, presence of numerous *Trichodina * attached to gill rakers, higher power of different views of *Trichodina* showing aboral disc with hook-like elements**,** some of them sending fibrin like a fibrin-like thread (arrow). At 370 × magnification, with a scale bar representing 50 µm, **A** at 750 × magnification, with a scale bar representing 20 µm **B** at 2300 × magnification, with a scale bar representing 10 µm **C** at 900 × magnification, with a scale bar representing 20 µm **D**

In the summer season (Fig. 8 A &B), the gills showed lamellar lifting with partial lamellar fusion, desquamated lamellar epithelial cells, interlamellar necrotic debris, and a few lamellar telangiectasias. In the winter season (Fig. 8 C&D), the gills showed lamellar clubbing with lamellar necrosis and desquamation, or coalescing lamellar hyperplasia with fusion along the lengths of some lamellae or at the lamellar tips, forming synechiae. In the spring season (Fig. 8 E&F), the gills showed focal to diffuse coalescing hyperplasia of the lamellar epithelium, causing fusion of secondary lamellae with focal complete loss of lamellar epithelium. In the autumn season (Fig. 8 G-J), gill rakers showed thickening and expansion of lamellar tissue with abundant lamellar telangiectasia, severe branchial congestion, and lamellar fusion with goblet cell hyperplasia. Additionally, diffuse marked distortion of normal gill architecture was characterized by stunted, fused, or complete lack of secondary lamellae with massive interlamellar detachment of the gill epithelium admixed with many cellular infiltrates. In SEM, the gills showed parasite-host interactions. Numerous *Trichodina* spp. are attached to gill rakers surrounded by many small, rounded lymphocytes, ruffled macrophage-like cells, and mucus globules. The higher power of the different views of *Trichodina* spp. shows an aboral disc with hook-like elements and its oral infundibulum and body reactions that consist of many cellular infiltrates, some of which send fibrin-like threads to attack the parasite. In addition, embedded disc-like bodies were attached to the gills and were admixed with many mucus globules and cellular infiltrates, with clumps of mucus globules and threads attached to the gill surface (Fig. 9 A-D).

### SEM examination

**Fig. 10:**
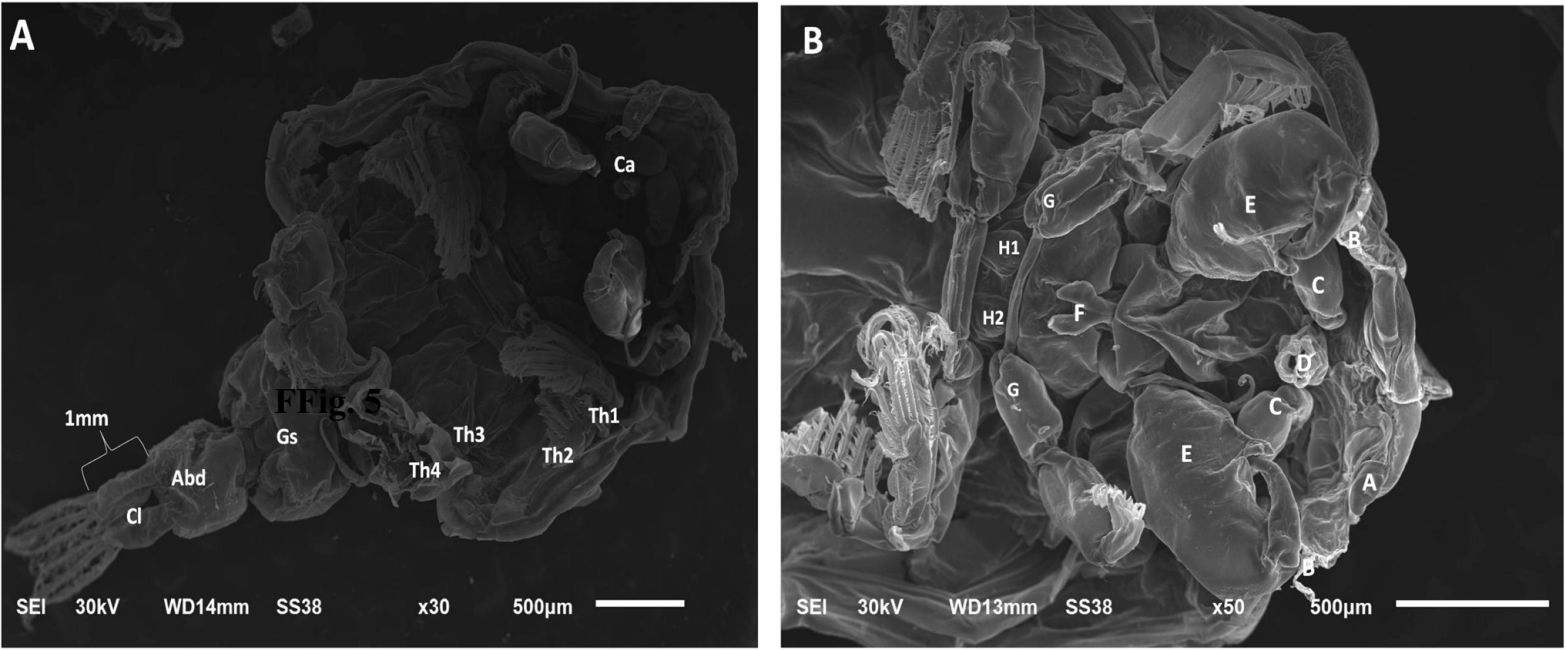
**A** Scanning electron micrographs of *Caligus spp*. Ventral view. Body areas. Magnification at 30X. Ca: Cephalic area, Th1: 1 st thoracopod, Th2: 2nd thoracopod, Th3: 3rd thoracopod, Th4: 4th thoracopod, Gs: genital segment, Abd: abdomen and Cl: caudal lamella. **B** *Caligus spp*. Ventral view. viewed under SEM. (Cephalothorax). A: lunule, B: antenna 1, C: antenna 2, D: mouth cone E: maxillae, F: sternal furca with tapering tines, G: leg 1, G: leg 2, H1: swelling 1 and H2: swelling 2. Magnification at 50X

Scanning Electron Microscopy revealed two main body regions: a broad anterior cephalothorax (Fig. 10A; Ca) and a posterior region composed of four thoracic segments, the genital segment (Fig. 10A; Gs), and the abdomen. The ventral surface of the cephalothorax displayed an anterior antennal region, with the first antennae (antennules, Fig. 10B; B) followed posteromedially by the second antennae (Fig. 10B; C). These structures flanked a prominent oral cone bordered by postoral processes (Fig. 10B; D). Posteriorly, maxillae (E), maxillipeds, and the sternal furca (F) formed the feeding and attachment apparatus. Additionally, the ventral surface bore two rounded swellings likely involved in adhesion, tipped with short setules (Fig. 10B; H1–H2), consistent with attachment functions in parasitic copepods.

**Fig. 11:**
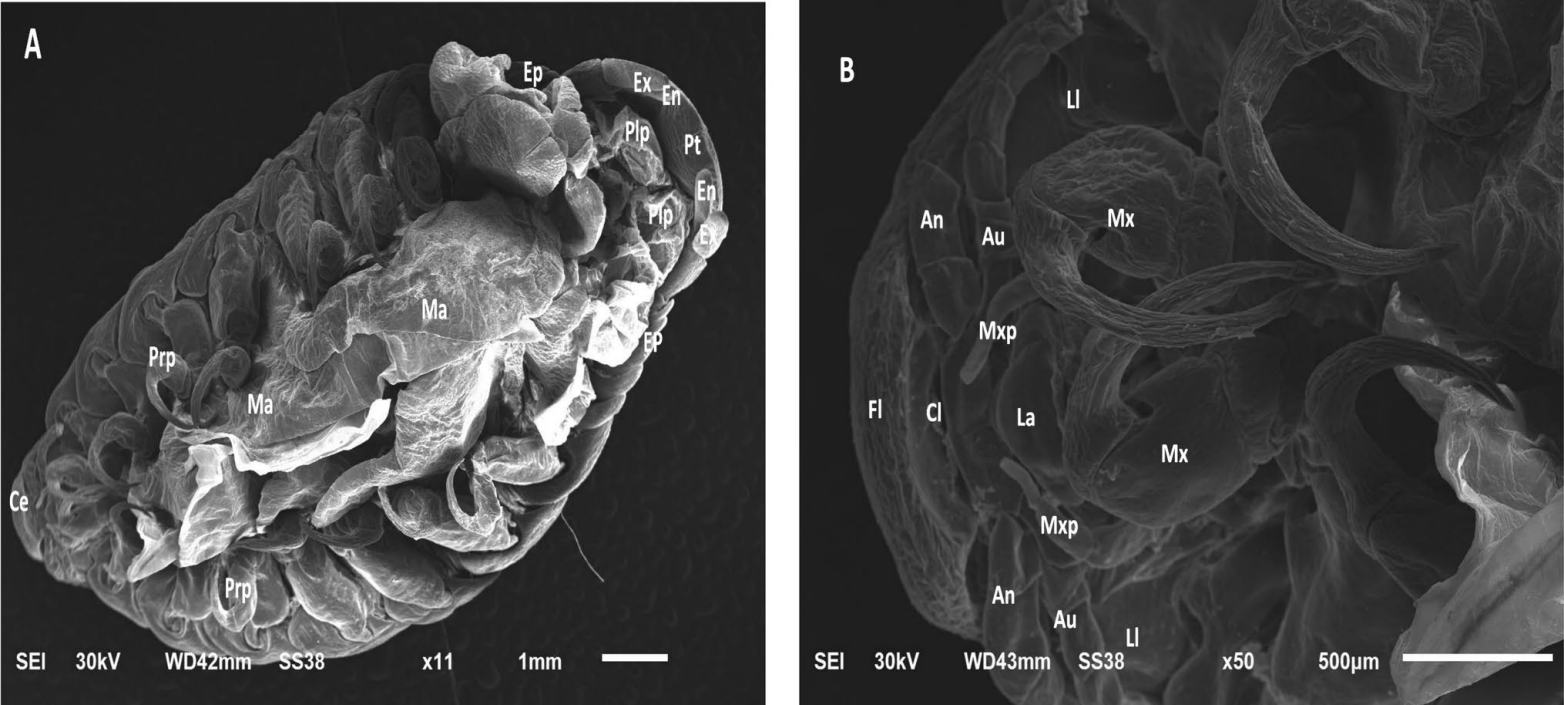
Scanning electron micrographs of presumptive *Livoneca redmanii* (Leach, 1818) recovered from European seabass (*Dicentrarchus labrax*). **A** Ventral view showing morphological features: Ce = cephalon; Ep = epimera; Prp = pereopods; Ma = marsupium; Plp = pleopods; Pt = pleotelson; En = endopodite of uropod; Ex = exopodite of uropod. Magnification = 11 ×. **B** Ventral anterior view highlighting mouthparts and sensory appendages: An = antenna; Au = antennule; Cl = clypeus; Fl = frontal lamina; La = labrum; Ll = lateral lamina; Mx = maxilla; Mxp = maxilliped. Magnification = 50 ×. Species-level identification was based only on morphological resemblance to published descriptions (Bruce 1986)

Scanning microscopic examination of *L. redmanii* (Leach, 1818) exhibited a body consisted of a non-segmented cephalon, a pereon, and a pleon. The pereon, comprised of seven articulating pereonites, had the largest body area (Fig. 11A). Each pereonite bore a pair of pereopods, facilitating locomotion and host attachment. The pleon carried pleopods, a pleotelson, and uropods, with the exopodites larger than the endopodites. The cephalon, exposed and triangular, included a concave frontal lamina, a convex clypeus, and lateral laminae enclosing sucking mouthparts (Fig. 11B). A marsupium (brood pouch) was evident on the female’s ventral surface. Here, the "pereon" in isopods is functionally and positionally equivalent to the "thorax" in copepods (*Caligus*), though composed of distinct articulating somites.

## Discussion

In Egypt, mariculture is highly significant and plays a major role in the aquaculture industry and in economic development (Abdel-Hady et al. 2024). Parasitic diseases in fish are a major source of economic loss in fish farming (Buchmann 2022). This study investigated the seasonal prevalence, diversity, and pathological effects of ectoparasites (*Trichodina* spp., *Caligus* spp., and *Isopoda* spp.) in marine fish cultured at a single coastal seabass (*D. labrax*) earthen ponds farm. The work also examined relationships between parasite occurrence, environmental parameters, and heavy metal concentrations. The findings highlight clear seasonal patterns and demonstrate how environmental fluctuations and water quality factors influence parasite dynamics and host health. The clinical findings observed in the fish farms indicated that naturally infected *D. labrax* exhibited respiratory distress and surface swimming with an open mouth. The other fish showed a hemorrhagic area in the abdomen. In some cases, Isopoda and *Caligus* species were observed by the naked eye on the gill and body surface, respectively. Infected gills were covered with an excessive mucous layer. These findings are consistent with those of previous studies (Noga 2010; El-Deen et al. 2013; Marzouk et al. 2019).

The morphological characteristics of the collected *Trichodina* spp. were similar to those described for comparable parasites isolated from Egyptian fish hosts such as *Oreochromis niloticus* (Aly et al. 2020; Attia et al. 2021), *Lates niloticus* (Hussein et al. 2012). Similar morphological characteristics of the *Caligus spp* we retrieved were also comparable to those of other *Caligus spp*. isolated from *M. cephalus* and *D. labrax* (Abdelsalam et al. 2025). Similarly, Isopoda found in this study had morphological characteristics similar to those previously described in other Egyptian fish hosts, including *D. labrax* (Abdallah and Hamouda 2022a, b), *Argyrosomus regius* (Fadel et al. 2020), *M. cephalus* (Helal and Yousef 2018), and *Tilapia Zillii* (Mohammed-Geba et al. 2019).

Parasite populations are known to fluctuate with seasonal shifts in water temperature, salinity, and the availability of suitable hosts. Our study recorded a high prevalence of ectoparasitic infestations (77%) in the examined seabass, which is inconsistent with the reported total infestation rates of (77.1%) and (71.25%) (Mahmoud et al. 2017; Ibrahim et al. 2024), In contrast, El-Deen et al. (2020), Shaheen et al. (2017) and El-Deen et al. (2015), reported infestation rates of (45.83%), (50.7%) and (35%) respectively, which may be attributed to the diversity of the regions studied, the ecological and environmental elements, and the investigation period. *Trichodina* is a genus of ciliated protozoan parasites that significantly affects the health of sea bass, particularly in aquaculture settings, inducing chronic mortality (Ihwan et al. 2016). Regarding the total prevalence of infestation, a higher infestation rate was recorded for *Trichodina* species in winter (92%) and infestation decreased in summer (48%). These findings are in line with those of Marzouk et al. (2019), who reported that all protozoan infestations (*Trichodina heterodentata, Epistylis, Amyloodinium ocellatum, and Microsporidia*) peaked in autumn and decreased in summer. These findings also suggest that the rate of protozoan infection is significantly affected by water quality fluctuations. Moreover, the seasonal prevalence of crustacean infestation was the highest in winter (*Caligus*, 60%) and summer (Isopoda, 8%) but not in spring and autumn. This finding could be attributed to a combination of environmental and biological influences. For instance, the sharp rise in *Caligus* infestations during winter may reflect conditions that favor parasite survival and reproduction. Colder temperatures could slow developmental processes, giving parasites more time to attach to fish. In contrast, spring and autumn may present less favorable scenarios due to moderate temperatures and stronger water currents, which can interfere with larval dispersal and survival (Pimentel-Acosta et al. 2023; Lepe-López et al. 2021). Furthermore, the fish’s immune defenses and competition between different parasite species may further explain why infestation rates remain low outside of the winter months (Ahmed et al. 2022; Tavares-Dias 2023). These findings are consistent with El-Deen et al. (2013), who found a high prevalence of infested marine seabass and mullet (91.6 and 71.3%, respectively) throughout the summer and their absence in the autumn and winter. According to Yalım et al. (2014), European sea bass *(D. labrax)* infested with *Caligus minimus* shows a progressive increase in parasite prevalence from autumn to winter. Our findings are in contrast with those obtained by Tadros et al. (2020), who found that copepods (71%) and Isopoda (69%) had their highest values in autumn and winter, respectively, and the lowest Isopods (29%) and copepods (19%) values were in spring and summer. Additionally, Eissa et al. (2017b) demonstrated that high levels of crustaceans were found in the spring (84%), followed by the summer (80%). These variations in prevalence could be caused by variations in the fish collection location (Eissa et al. 2017b), immune response of fish at various temperatures (Antonelli et al. 2016; Khalil et al. 2018), or the plankton and food composition of *D. labrax* during different seasons (Tekin-Özan et al. 2008).

Fish gills are extremely sensitive organs in charge of respiration, preserving the optimum osmotic pressure and acid–base equilibrium of internal fluids. They play an essential role in promoting food drainage and eliminating harmful and extra metabolic products (Strzyzewska et al. 2016). In the present study, different characteristics were observed using both light microscopy and SEM, and the gills showed different histopathological lesions owing to parasitism. Gills showed parasite-host interactions and numerous *Trichodinids* attached to gill rakers surrounded by many small, rounded lymphocytes, ruffled macrophage-like cells, and mucus globules. The extensive damage to the branchial tissue demonstrated the parasite's invasiveness and ability to harm the host tissue; the SEM findings were consistent with the findings of (Maged et al. 2024). As well as a higher power of different views in SEM of *Trichodina* spp. showed aboral disc with hook-like elements and its oral infundibulum and the body reactions that consisted of many cellular infiltrates, some of them sending fibrin-like threads to attack the parasite, the SEM findings align with the findings described elsewhere (Islas-Ortega and Aguilar-Aguilar 2014; Ortega 2019), which suggest an active immune response aimed at combating the parasite. This damage could be attributed to the attachment nature of each parasite, which results in mucus oversecretion and lamellar hyperplasia with secondary lamellar fusion (Paul and Sahoo 2024). Similar results were observed in a study conducted by Mahmoud et al. (2017), who observed excessive mucus secretion, paleness, hyperplasia, and necrosis of epithelial cells in primary gill filaments of wild and cultured marine fish in Egypt. Similarly, Maged et al. (2024) reported massive destruction of lamellar epithelium with necrotic lamellar epithelial debris, extensive lamellar desquamation, and lamellar lifting with partial-to-complete lamellar fusion. Ali and Ismail (2022) observed significant mononuclear cell infiltration, and subsequent lamellar erosion and necrosis. Additionally, hyperplasia, necrosis of the gill filament epithelium, and inflammatory responses can lead to gill tissue damage (Adawy et al. 2016).

The SEM-documented features in *Caligus* spp., particularly the sturdy sternal furca, antennae, and maxillipeds, coincide previous studies underscore their function in securely attaching to host gill epithelia, causing histological damage like hemorrhage and hyperplasia (Abdelsalam et al. 2024; El-Aziz et al. 2014). Likewise, the shape of *L. redmanii*, including uropods and suction-adapted mouthparts, matches descriptions by Abdallah and Hamouda (2022a, b), who noted epithelial erosion and lamellar sloughing. These mechanical features reflect host-specific disease processes, supported by Korun and Tepecik (2005) and Mladineo et al. (2020), where parasite attachment resulted in localized necrosis and inflammation.

Aquatic environments and fish health are closely interconnected, and both absorb a range of pollutants that adversely affect the well-being of resident species and jeopardize their survival (Ali et al. 2020). There is growing awareness that parasitism needs to be studied under environmental conditions of the environment (Guitard et al. 2022). Water pollution can significantly limit the prevalence of some fish parasite species and alter their qualitative and quantitative structures by affecting their eggs, free-living larval stages, and intermediate or final hosts (Ashmawy et al. 2018). The findings of the current study showed that the salinity, pH, and temperature readings and seasonal variations in water temperature ranged from a minimum of 18.5 °C in the winter to a maximum of 27 °C in the summer. Abdel-Halim and Aly-Eldeen (2016) note a similar pattern. This study found that pH ranged from 8.1 to 8.5, with winter recording the highest value and spring the lowest value. The amount of sewage pollution and photosynthesis by algae affects the pH level. Furthermore, because of the high concentrations of organic contaminants and the outflow of brackish water, lower pH values are associated with lower oxygen concentrations (Abdel-Halim and Aly-Eldeen 2016). These results concurred with those of Ojwala et al. (2018), who noted that water slightly affects parasite abundance at alkaline pH. According to Xia et al. (2021), salinity reflects the level of contamination in aquatic environments by demonstrating the rate at which land water discharges dilute saltwater. Salinity in this study had the highest value of 41.85 g/L in winter, as in a study by Elmorsi et al. (2017), who found that the salinity percentage was greater in winter along the Mediterranean Sea coast in Egypt.

Our study recorded a correlation analysis, which demonstrated a very strong negative and statistically significant relationship between *Trichodina* prevalence and water temperature (*r* = −0.99, *P* = 0.003, *R*^2^ = 99%). This suggests that lower temperatures were associated with higher infestation rates. The strength and significance of this correlation indicate that temperature may be a critical environmental factor influencing *Trichodina* proliferation. However, it should be noted that our correlation was based on only four seasonal data points representing aggregated data, which may still limit the statistical strength of the result and increase the risk of overinterpreting the correlation. Our findings are consistent with the regression study conducted by Gaze (1995), which revealed statistically significant negative relationships between water temperature and the infestation of *Trichodina domerguei* and *Trichodina tenuidens*, as temperature affects the morphological traits of the adhesive discs of these *Trichodinid* species. In addition, the prevalence and mean densities of *Trichodina* dynamics in *Merlangius merlangus* were strongly influenced by water temperature, showing seasonal turnover in December when the water temperature declined to 8 °C and exhibiting a robust correlation (*R*^2^ = 89%) (Ogut and Palm 2005). Conversely, others have demonstrated that temperature variations can profoundly affect life cycles and transmission dynamics (Wharton 1999). The highest prevalence of *Trichodina* spp was reported during summer when the water temperature was 31.5 °C. In contrast, the lowest disease prevalence was recorded in winter, indicating a positive correlation between water temperature and *Trichodina* infestation rates (Soliman et al. 2013).

In contrast, a strong negative but non-significant correlation was observed between *Trichodina* prevalence and salinity level (*r* = −0.75, *P* = 0.250, *R*^2^ = 56%). Although the direction of this relationship implies that higher salinity may reduce parasite prevalence, the lack of statistical significance suggests that this association may be influenced by other environmental or biological factors. Mello et al. (2015) found no significant changes in the occurrence or intensity of *Rhabdosynochus rhabdosynochus* in the gills of the sea bass (*Centropomus undecimalis)* at different salinity levels (15 and 32 ppt). This could be related to the biological properties of arrow bass, which is a euryhaline and diadromous species and, like its host, *R. rhabdosynochus*, may have acquired salinity tolerance through coevolutionary mechanisms (Jerônimo et al. 2022).

(Hg), Cd (Cd), Fe (Fe), Pb (Pb), Zn (Zn), Cu (Cu), and As (As) concentrations were significantly higher than the upper limits. High concentrations of these metals may result from human activities, industrial processes, or harm to aquatic organisms. These findings are consistent with those reported by Mahmoud et al. (2019), who stated that several types of pollution, including human activity, industrial processes, marine transportation, and fisheries, are pushed through both water surfaces. This finding supports that of Amoatey and Baawain (2019), who claimed that long-term exposure to water pollutants, even at low concentrations, might render aquatic animals more vulnerable to a range of diseases by interfering with their immune, reproductive, and developmental systems. Furthermore, many heavy metals can enter surface water through the discharge of treated and untreated liquid wastes. Similarly, Tytła (2019) and Mahmoud et al. (2015) studied the most crucial factors in determining the possible harm that heavy metals can cause to the environment and, consequently, to living organisms. They found that sewage sludge generated in a specific wastewater treatment plant was one of the primary sources of heavy metal pollution.

### Study limitations

In the current study, using just one marine fish farm may limit how well the results apply to other farms with different environments or management styles. Although more frequent sampling could have offered better insights into changes in heavy metal levels over time, budget limitations made that difficult. Still, the farm followed steady management routines, and the environmental conditions stayed fairly consistent throughout the study. We recognize these aspects as limitations and suggest future studies cover multiple locations and seasons to better understand regional and seasonal differences. Additionally, using molecular tools to identify and genotype parasites, along with broader toxicological testing, could shed more light on how environmental pollutants influence parasite outbreaks in farmed fish.

## Conclusion

This study examined ectoparasitic infections as bioindicators of the environmental and anthropogenic influences on marine fish farms. A High prevalence (77%) was recorded for *Trichodina* spp. (75%), *Caligus spp*. (15%), and Isopoda spp. (2%). *Trichodina* was negatively correlated with temperature and salinity. The prevalence of parasites was associated with water quality and heavy metal concentrations, corroborating the utility of parasites as indicators of environmental stress. Regular monitoring of water quality in Egyptian aquaculture is crucial to prevent contamination and reduce the spread of parasites.

## Data Availability

All data supporting the findings of this study are available within the paper.
